# Identification of *MYLK3* mutations in familial dilated cardiomyopathy

**DOI:** 10.1038/s41598-017-17769-1

**Published:** 2017-12-13

**Authors:** Takashige Tobita, Seitaro Nomura, Hiroyuki Morita, Toshiyuki Ko, Takanori Fujita, Haruhiro Toko, Kenta Uto, Nobuhisa Hagiwara, Hiroyuki Aburatani, Issei Komuro

**Affiliations:** 1 Department of Cardiology, Tokyo Women’s Medical University, Tokyo, Japan; 2 Department of Cardiovascular Medicine, Graduate School of Medicine, The University of Tokyo, Tokyo, Japan; 3 Genome Science Division, Research Center for Advanced Science and Technology, The University of Tokyo, Tokyo, Japan

## Abstract

Dilated cardiomyopathy (DCM) is a primary cause of heart failure, life-threatening arrhythmias, and cardiac death. Pathogenic mutations have been identified at the loci of more than 50 genes in approximately 50% of DCM cases, while the etiologies of the remainder have yet to be determined. In this study, we applied whole exome sequencing in combination with segregation analysis to one pedigree with familial DCM, and identified a read-through mutation (c.2459 A > C; p.*820Sext*19) in the myosin light chain kinase 3 gene (*MYLK3*). We then conducted *MYLK3* gene screening of 15 DCM patients (7 familial and 8 sporadic) who were negative for mutation screening of the previously-reported cardiomyopathy-causing genes, and identified another case with a *MYLK3* frameshift mutation (c.1879_1885del; p.L627fs*41). *In vitro* experiments and immunohistochemistry suggested that the *MYLK3* mutations identified in this study result in markedly reduced levels of protein expression and myosin light chain 2 phosphorylation. This is the first report that *MYLK3* mutations can cause DCM in humans. The clinical phenotypes of DCM patients were consistent with *MYLK3* loss-of-function mouse and zebrafish models in which cardiac enlargement and heart failure are observed. Our findings highlight an essential role for cardiac myosin light chain kinase in the human heart.

## Introduction

Dilated cardiomyopathy (DCM) is a genetic disorder that causes heart failure, life- threatening arrhythmias, and cardiac death^1^. Familial DCM accounts for 20–35% of all cases^1^, and mutations in more than 50 genes are known to cause the development of DCM^2^; however, the etiological causes remain unidentified in approximately 50% of DCM cases. Recently, whole exome sequencing (WES) has been used successfully to identify genetic abnormalities in relatively rare hereditary diseases^3^.

Cardiac myosin light chain kinase (cMLCK) encoded by the myosin light chain kinase 3 (*MYLK3*) phosphorylates cardiac myosin light chain 2 (MLC2, encoded by *MYL2*) and facilitates actin−myosin interactions that enhance contractile force in the animal heart^4–6^. In neonatal rat cardiomyocytes, overexpression of *MYLK3* promoted sarcomere assembly, while knockdown disrupted sarcomere formation^5^. Furthermore, *MYLK3* loss-of-function in mice and zebrafish induced cardiac dysfunction and heart failure^4,7^, suggesting that this kinase is indispensable for cardiac homeostasis.

In this study, we report the first DCM cases harboring *MYLK3* mutations identified using WES. Sequencing analyses of unrelated DCM patients revealed an additional *MYLK3* frameshift mutation. Our human genetic analysis suggests that *MYLK3* is a novel pathogenic gene for DCM.

## Results

### Study population

**Figure 1:**
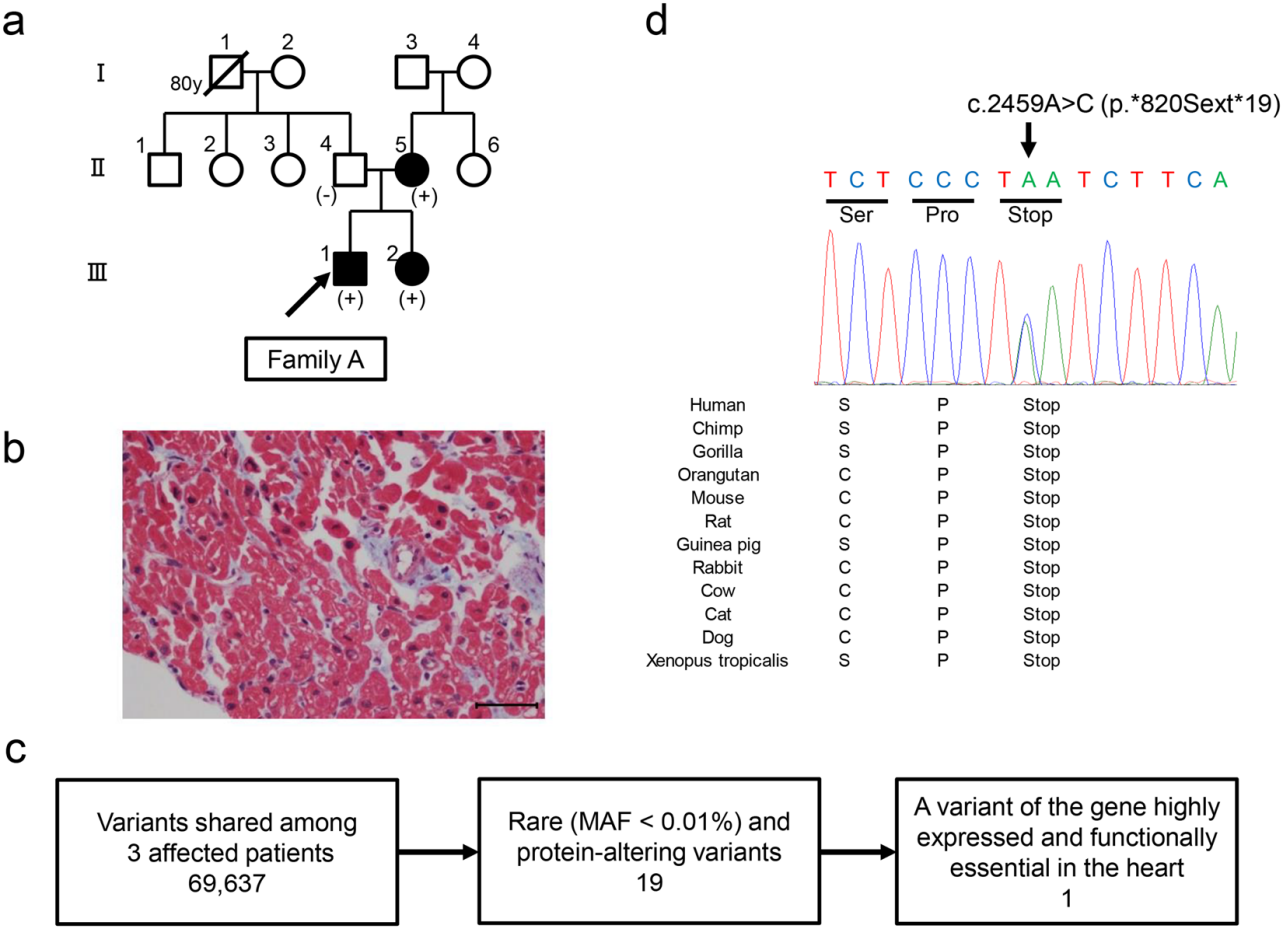
WES analysis identified a read-through mutation in the myosin light chain kinase 3 gene (*MYLK3*) in all three affected individuals. (**a**) Familial pedigree of DCM patients analyzed by whole exome sequencing. Squares and circles represent males and females, respectively. A slash represents a deceased individual. Black-filled symbols indicate patients with a clinical diagnosis of DCM. Open symbols represent unaffected family members. The proband is indicated by an arrow. *MYLK3* mutation carriers are indicated by a plus sign. (**b**) Endomyocardial biopsy findings of the proband revealed myocardial changes and fibrosis (Masson trichrome stain; scale bar, 50 µm). (**c**) Summary of variant filtering. The number of variants is described in each category. MAF, minor allele frequency. (**d**) Sanger sequencing analysis verified the presence of a *MYLK3* read-through mutation in family A. The stop codon affected by this mutation is well conserved across evolution.

A 16-year-old male (family A-III-1; Fig. 1a) diagnosed with DCM at 10 months of age was started on β-blocker and angiotensin-converting enzyme (ACE) inhibitor therapy. An endomyocardial biopsy of the proband was consistent with DCM and showed mild fibrosis (Fig. 1b). He exhibited substantially improved left ventricular (LV) function after a few years of treatment. The sister of the proband (family A-III-2; Fig. 1a) was diagnosed with DCM at 9 months of age. She was also started on β-blocker and ACE inhibitor therapy, and exhibited improved LV function after the initiation of treatment. The mother of the proband (family A-II-5; Fig. 1a) was diagnosed with DCM at 40 years of age. She showed relatively mild symptoms and a later disease onset compared to her children. The father of the proband (family A-II-4; Fig. 1a) had no symptoms of heart failure and echocardiography showed normal cardiac contraction.

### WES of the three affected patients with familial DCM

**Figure 2:**
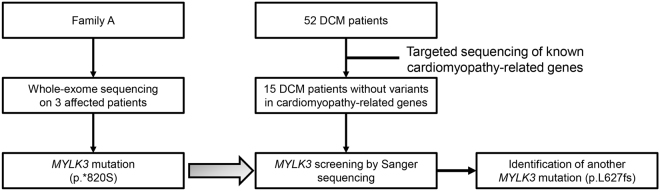
Study flowchart.

The inheritance pattern was thought to be autosomal dominant, and we performed WES on the three affected patients in family A (Figs 1a and 2). The median read depth in the targeted exonic region was 91 × , and 96.3% of the targeted exonic regions had over 20 × read depth. Variant filtering was conducted as shown in Fig. 1c, and yielded 19 rare variants that potentially affected protein function (Supplementary Table 1). We found no shared mutations in genes that were previously reported to be associated with DCM. Then, we reviewed the variants according to the guidelines for implicating sequence variants^8^. Among the genes, *MYLK3* was highly expressed and functionally established in the heart, and was considered to be the only candidate likely to cause DCM. The variant was a read-through mutation (c.2459 A > C; p.*820Sext*19) resulting in a protein product with a 19-amino acid C-terminal extension not present in any population database. The variant was predicted to be deleterious based on its combined annotation-dependent depletion (CADD) score (18.79). Sanger sequencing confirmed that the variant was present in the three affected patients (Fig. 1d, Supplementary Fig. 1), but not in the unaffected father of the proband (family A-II-4; Fig. 1a), indicating that the variant segregated with the disease phenotype.

### MYLK3 genetic screening

**Figure 3:**
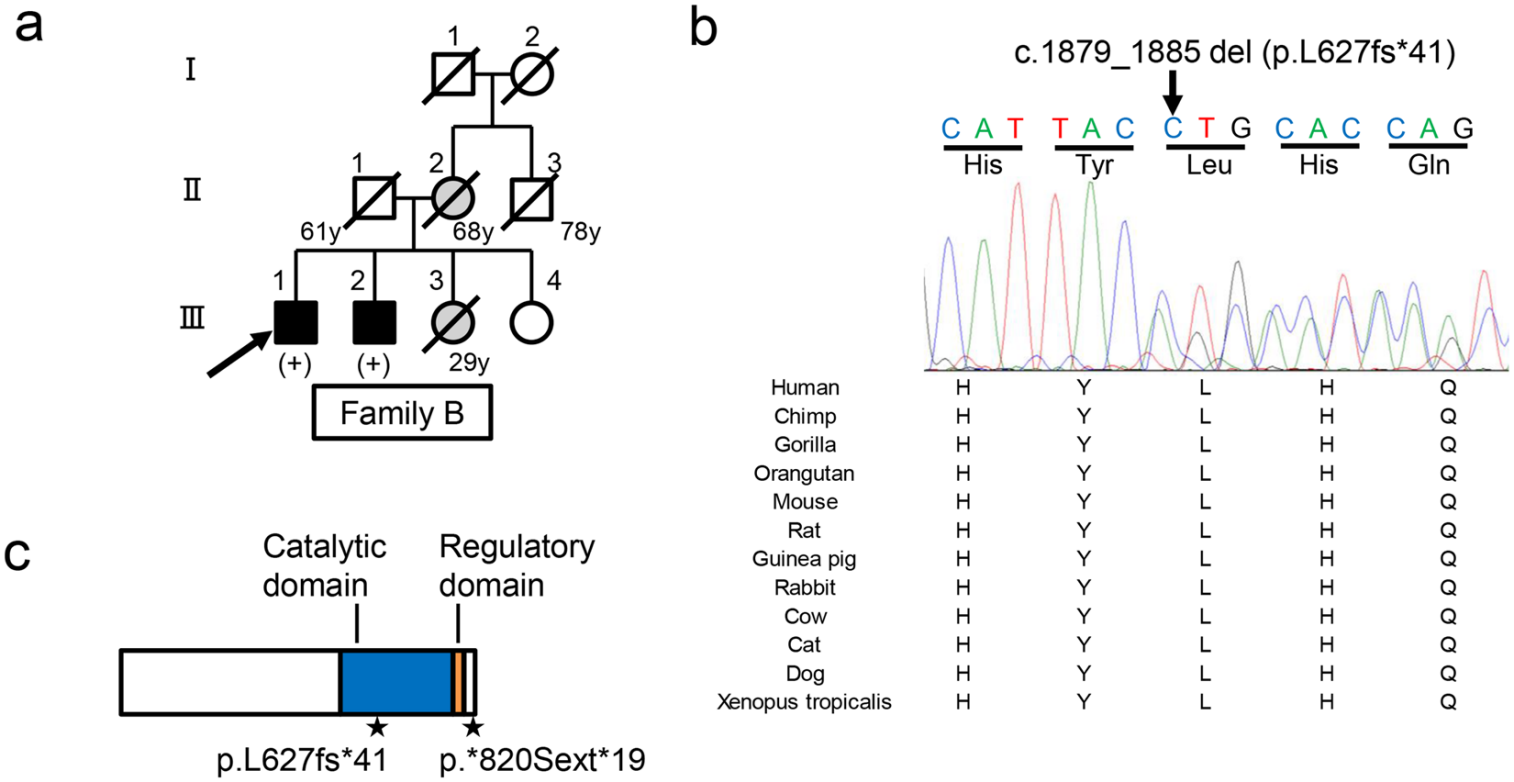
*MYLK3* gene sequencing analysis revealed a frameshift *MYLK3* mutation in family B. (**a**) Familial pedigree of DCM patients harboring a *MYLK3* frameshift variant. Squares and circles represent males and females, respectively. Slashes represent deceased individuals. Black-filled symbols indicate patients with a clinical diagnosis of DCM. Gray-filled symbols denote a suspicion of DCM. Open symbols represent unaffected family members. The proband is indicated by an arrow. *MYLK3* mutation carriers are indicated by a plus sign. (**b**) Sanger sequencing analysis verified the presence of a *MYLK3* frameshift mutation in family B. The amino acid residue altered by this mutation is well conserved across evolution. (**c**) *MYLK3* domain structure. The stars represent the positions of the mutations identified in this study.

To examine whether other *MYLK3* variants cause DCM, we first conducted targeted sequencing in our 52-patient unrelated DCM cohort and identified 15 patients (8 familial and 7 sporadic) with uncharacterized genetic etiology in known cardiomyopathy-related genes (Fig. 2, Supplementary Table 2). *MYLK3* gene sequencing in these 15 patients identified an additional unique frameshift variant (c.1879_1885del; p.L627fs*41) in one familial DCM patient (family B-III-1; Fig. 3a and b). This frameshift was also predicted to be deleterious (CADD score of 35), resulting in a premature stop codon at E667 that likely disrupts both the catalytic and regulatory domains of the protein (Fig. 3c). Familial analysis revealed that the affected brother of the family B proband (family B-III-2; Fig. 3a) harbored the same variant. The proband and his brother were diagnosed with DCM at 56 and 52 years of age, respectively. Their cardiac function was improved after the initiation of treatment. One of their sisters (family B-III-3; Fig. 3a) had complained of dyspnea, but died in her twenties without an accurate diagnosis, so a DNA sample and detailed clinical information were not available. The mother of the proband (family B-II-2; Fig. 3a) also complained of dyspnea and palpitation, but her detailed clinical information and the presence or absence of DCM were also unclear. The father of the proband (family B-II-1; Fig. 3a) had no symptoms. We then performed WES on the two affected patients in family B and conducted variant filtering as described above to confirm the absence of additional pathogenic variants shared by these two affected patients (Supplementary Table 3).

Overall, we identified two *MYLK3* mutations in five DCM patients. The clinical characteristics of these patients are summarized in Table 1. Two patients (both in family A) developed severe DCM in infancy and the other three (one in family A and two in family B) developed adult-onset DCM. Life-threatening arrhythmia was not seen in any case. All of the patients with *MYLK3* mutations identified in our study showed an improvement of cardiac function after the initiation of medical therapy.

**Table 1:** Clinical features of five DCM patients harboring *MYLK3* mutations.

Subject	Sex	Age of diagnosis	Presenting symptoms	Arrhythmia	Pathology (heart biopsy)	Cardiac function at onset	Age at follow-up	Present cardiac function	Device implantation	Outcome
**Family A**										
II-5	W	40 y	dyspnea, heart failure	NSR	mild myocardial change and fibrosis	LVEF = 40%, LVEDD 52 mm, LVESD 35 mm	42 y	LVEF = 56%, LVEDD 42 mm, LVESD 30 mm	None	alive
III-1	M	10 m	cough, poor weight gain	NSR	moderate myocardial change and mild fibrosis	LVEF = 13%, LVEDD 59 mm, LVESD 53 mm	17 y	LVEF = 44%, LVEDD 53 mm, LVESD 39 mm	None	alive
III-2	W	9 m	cough, poor weight gain	NSR	moderate myocardial change and mild fibrosis	LVEF = 7%, LVEDD 43 mm, LVESD 39 mm	13 y	LVEF = 23%, LVEDD 49 mm, LVESD 44 mm	None	alive
**Family B**										
III-1	M	56 y	dyspnea, heart failure	AF	NA	LVEF = 38%, LVEDD 74 mm, LVESD 60 mm	66 y	LVEF = 43%, LVEDD 59 mm, LVESD 51 mm	None	alive
III-2	M	52 y	dyspnea, heart failure, palpitation	AF	moderate myocardial change and mild fibrosis	LVEF 20–30%, LV enlargement	64 y	LVEF = 51%, LVEDD 44 mm, LVESD 31 mm	None	alive

Abbreviations: LV, left ventricular; EF, ejection fraction; EDD, end-diastolic diameter; ESD, end-systolic diameter; NSR, normal sinus rhythm; AF, atrial fibrillation; NA, not applicable.

### Western blot analysis using MYLK3 constructs

**Figure 4:**
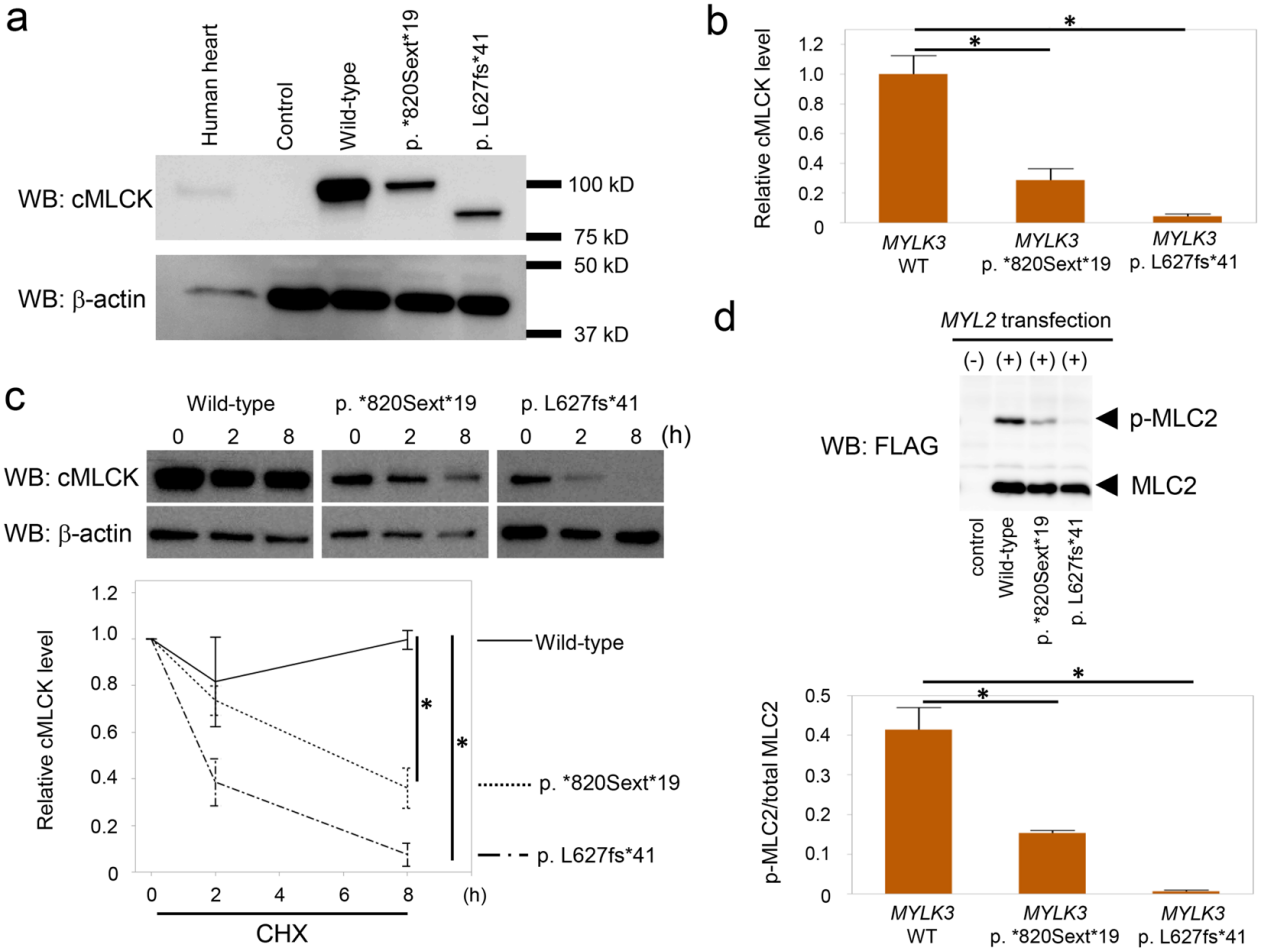
Western blot analysis suggested decreased expression levels of mutant cMLCK and MLC2 phosphorylation. (**a**) Expression of wild-type and mutant cMLCK protein in transfected HEK293T cells. Western blot analysis showed the reduced expression of mutant cMLCK protein. The blots were run under the same experimental conditions. The uncropped images are in Supplementary Fig. 2. (**b**) Densitometric quantification of western blots. Data are expressed as mean ± SEM (N = 3). *P < 0.05. (**c**) Wild-type or mutant MYLK3 transfected HEK293T cells were treated with cycloheximide (CHX) for indicated times. Western blot analysis showed rapid reduction of mutant proteins. Data are expressed as mean ± SEM (N = 3). *P < 0.05. The blots were run under the same experimental conditions. The uncropped images are in Supplementary Fig. 3. (**d**) Phos-tag SDS-PAGE followed by western blot analysis showed phosphorylated and non-phosphorylated forms of MLC2 using anti-FLAG antibody. Levels of phosphorylated MLC2 were remarkably reduced when co-transfected with mutant *MYLK3* and *MYL2* constructs. The ratio of phosphorylated MLC2 to total MLC2 (phosphorylated MLC2+ non-phosphorylated MLC2) was determined. Data are expressed as mean ± SEM (N = 3). *P < 0.05. The uncropped images are in Supplementary Fig. 4.

We examined the effects of both *MYLK3* mutations on protein expression. We transfected HEK293T cells with either wild-type or mutant *MYLK3* (p.*820Sext*19 and p.L627fs*41) complementary DNA (cDNA) constructs. Western blot analysis using an antibody against residues 27–144 of human MYLK3 revealed significantly reduced mutant protein expression levels compared to the wild-type protein (p.*820Sext*19: 28.6% ± 7.8%, p.L627fs*41: 4.3% ± 1.7%; Fig. 4a and b). Cycloheximide (CHX) chase experiments revealed that mutant proteins were more rapidly degraded compared to the wild-type proteins, suggesting the instability of mutant proteins (Fig. 4c).

### Analysis of MLC2 Phosphorylation

We performed Phos-tag^9^ SDS-PAGE followed by western blot analysis to determine the activity of mutant proteins on MLC2 phosphorylation. We co-trasnfected HEK293T cells with constructs of either wild-type or mutant *MYLK3* and *MYL2* with Myc-FLAG-Tag at C-terminal. Western blot analysis using an anti-FLAG antibody revealed that MLC2 phosphorylation levels were significantly reduced when co-transfected with mutant *MYLK3* as compared with wild-type *MYLK3* (Fig. 4d).

### Immunohistochemistry of heart tissue

**Figure 5:**
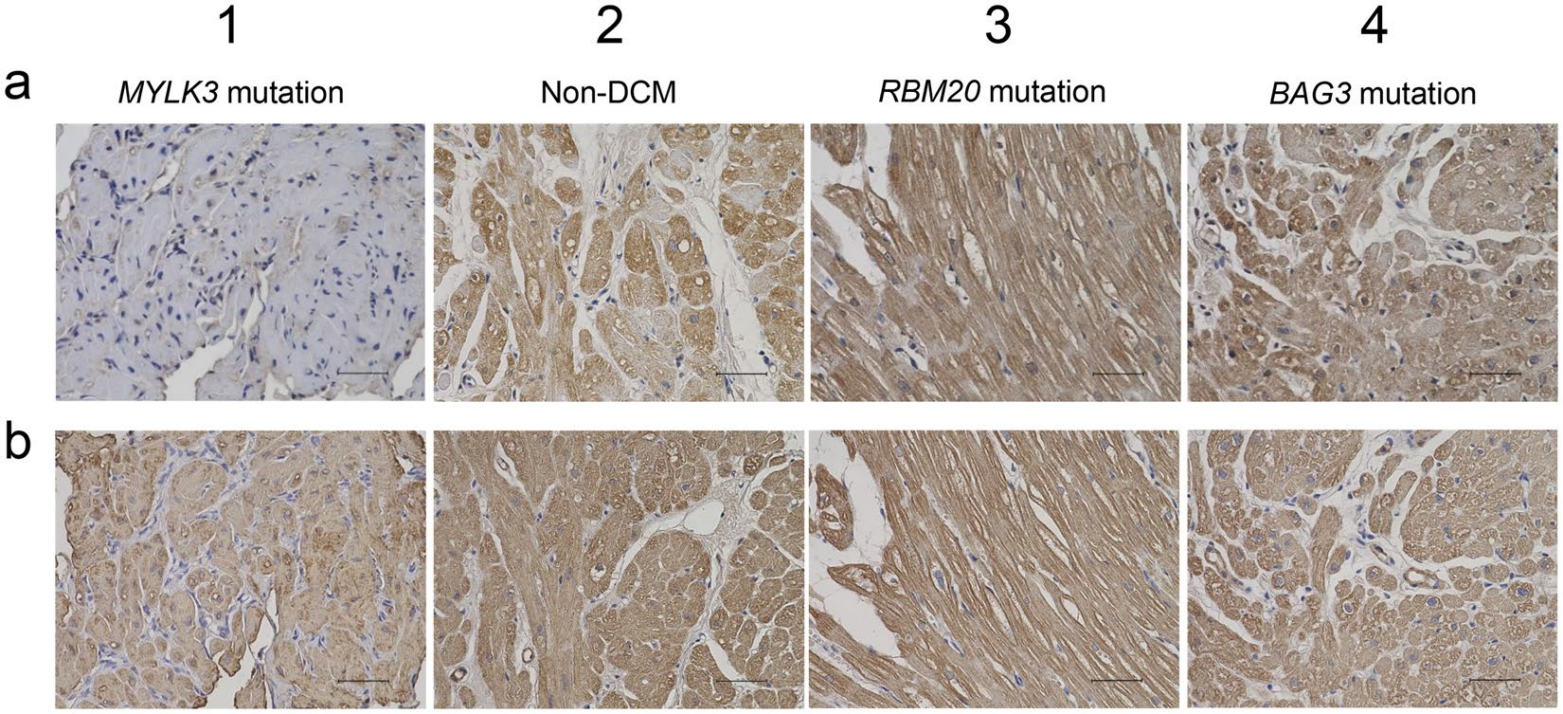
Immunohistochemistry suggested decreased expression levels of cMLCK. (**a**) Expressions of cMLCK protein were examined in heart tissue by immunohistochemistry (scale bar, 50 µm.). Column 1 represents cardiac tissue from the proband of family A. Column 2 represents cardiac tissue from individual without cardiac disorders. Column 3 and 4 represents cardiac tissue from a DCM patient harboring an *RBM20* mutation (without a *MYLK3* mutation) and a *BAG3* mutation (without a *MYLK3* mutation), respectively. (**b**) Detection of actin protein in heart tissue. Expression levels of actin were comparable in all subjects.

We performed immunohistochemistry of heart tissue from the patients to validate the effects of *MYLK3* mutation on protein expression. The antibody against residues 27–144 of human cMLCK showed a strong and uniform staining pattern in the cardiomyocytes of a non-DCM patient or a DCM patient harboring an *RBM20* mutation or a *BAG3* truncation mutation in our cohort. However, a tissue from the proband in family A with the *MYLK3* read-through mutation (p.*820Sext*19) showed significantly reduced cMLCK protein levels (Fig. 5a), although actin expression levels were comparable in all subjects (Fig. 5b).

### Variants shared by early-onset DCM siblings in family A

Since the two siblings in family A showed an early-onset and severe DCM phenotype compared to the other three affected patients harboring *MYLK3* mutations, we further analyzed whether the only two siblings in family A shared a second-hit variant. After variant filtering in the WES data, we found 14 rare variants that potentially affected protein function (Supplementary Table 4). Among these variants, a filamin C (*FLNC*) nonsense mutation (c.1444 C > T; p.R482*) was most likely to contribute to the severe phenotype because recent studies have suggested an association between *FLNC* truncation mutations and DCM^10,11^. Sanger sequencing confirmed that the variant was inherited from their unaffected father.

## Discussion

In this study, we identified two mutations in *MYLK3* that were associated with human DCM pathogenesis. WES analysis identified candidate pathogenic mutations in three affected individuals within one pedigree, and led to the discovery of a *MYLK3* read-through mutation. Subsequent *MYLK3* gene sequencing analysis revealed a *MYLK3* frameshift mutation responsible for DCM in another family. Although the importance of cMLCK for proper cardiac function is strongly suggested by animal cardiomyopathy models *in vivo* and experimental data *in vitro* ^4,5,7^, no *MYLK3* mutation has been identified as a pathogenic factor of human cardiomyopathy. Our present findings strongly suggest that *MYLK3* mutations cause DCM, which emphasizes a pivotal role for cMLCK in human cardiac function.

The clinical characteristics of these five patients suggest that *MYLK3*-related DCM responds to pharmacological therapy and exhibits reverse remodeling. LV ejection fraction or end-diastolic diameter of all patients with *MYLK3* mutations in this study improved significantly after the initiation of treatment. Responsiveness to medical therapy is associated with the prognosis of DCM^12^. None of the *MYLK3*-related DCM patients developed a life-threatening arrhythmia or required heart transplantation during follow-up. These findings suggest that the progression of *MYLK3*-related DCM could be slowed or prevented by appropriate medical treatment.

The two novel mutations in *MYLK3* identified in this study were not present in any database and both were predicted to be deleterious *in silico*. One was a read-through mutation that causes the continued translation of the mRNA into the 3′-untranslated region, resulting in the production of an aberrant protein with a 19-amino acid extension. mRNAs lacking a stop codon and C-terminally extended proteins can be degraded more easily than wild-type molecules through a mechanism called non-stop decay or protein instability, leading to low protein expression^13–16^. In fact, more than 20 read-through mutations have been reported to cause inherited diseases^15^ and, for example, a case of heterozygous read-through mutation causing remarkable reduction of normal protein was reported^17^. These observations suggest that the read-through mutation identified in this study would not only create a simple extended protein but also would affect cMLCK protein expression levels. The other mutation was a frameshift predicted to create a premature stop codon, leading to nonsense-mediated decay or truncation with a disrupted catalytic domain (Fig. 3c). Western blot analysis revealed decreased expression levels of both mutant proteins compared to wild-type proteins (Fig. 4a and b) and CHX treatment showed instability of both mutant proteins (Fig. 4c). Phos-tag SDS-PAGE in combination with western blot analysis revealed the kinase activity of mutant proteins were lower than wild-type proteins (Fig. 4d). Furthermore, expression levels of cMLCK were significantly lower in the heart tissue from the proband of family A compared with that from the other DCM patients without a *MYLK3* mutation or non-DCM patient (Fig. 5a). Therefore, we suggest that these mutations result in decreases of expression levels and activity of cMLCK.

*MYLK3* hypomorphic mice exhibited reduced cMLCK expression and MLC2 phosphorylation as well as mild heart failure and fibrosis with a clinical course resembling human DCM, while cardiac development and sarcomeric maturation were normal^7^. Animal studies have shown that cardiac MLC2 phosphorylation increases actin−myosin interactions and cardiac contractile strength through the regulation of myosin head position, lever-arm stiffness, and myosin step-size^18,19^. MLC2 phosphorylation is reportedly reduced in end-stage heart failure^20^. These findings suggest that MLC2 phosphorylation by cMLCK is essential for normal cardiac function, and that the loss of cMLCK causes LV dysfunction and enlargement, consistent with our results.

Age of onset and disease severity varied substantially among our study patients, as in most previously reported cases of familial DCM^21,22^. Two siblings in family A developed DCM in infancy and the proband’s sister with suspected DCM in family B died in her twenties. Additionally, the two childhood-onset DCM patients in family A had severely impaired cardiac function at diagnosis. In this way, a wide spectrum of clinical features can be present^21^. This heterogeneity complicates the assessment of familial DCM. Previous studies have demonstrated that multiple variants in cardiomyopathy-related genes can be present in patients with DCM or other cardiomyopathies^23,24^. In these cases, patients harboring multiple variants are predisposed to early-onset and severe clinical phenotypes compared with those of other family members harboring only a single variant. In this study, we identified an additional *FLNC* nonsense mutation in two early-onset and severe DCM siblings of family A and this variant was inherited from their unaffected father. Reinstein *et al*. reported an early-onset DCM case with *FLNC* compound heterozygous mutations^11^. In their report, all relatives with only a nonsense mutation in the *FLNC* gene were completely normal as was the unaffected father in our family A, and an additional missense mutation was necessary to cause early-onset DCM. These findings exactly correspond to our observation that the coexistence of the *MYLK3* and *FLNC* mutations could contribute to the early-onset and severe phenotype of DCM, highlighting the possibility of oligogenic effects on DCM. Furthermore, *MYLK3* knockout mice have been reported to show a mild contractile abnormality under basal conditions and exhibit severe heart failure under cardiac stress^25^. Therefore, we consider that additional stresses including second-hit mutations could explain the heterogeneity of *MYLK3*-related DCM phenotypes, although further functional studies are required to prove this hypothesis.

Our results suggest that *MYLK3* functions as the causal gene of DCM in two unrelated families, but there are some limitations in this study. First, the number of cases, especially of heart samples is limited. We assessed expression levels of cMLCK semi-quantitatively by immunohistochemical staining but not quantitatively by western blot analysis because there is not sufficient amount of heart tissues. Since the biopsy sample could not be obtained from any family members, immunohistochemical staining was performed only for the proband. It is also difficult to conclude that reduced expression levels of cMLCK are due to mutation or secondary to heart failure, since we did not examine expression levels of cMLCK in the heart of patients with mutation who do not show heart failure. Expression levels of mutated cMLCK were significantly lower than those of wild type cMLCK in our transfection experiments (Fig. 4a) and expression levels of cMLCK were well-preserved in DCM patients harboring an *RBM20* mutation or a *BAG3* mutation (Fig. 5). Although these results suggest that the reduced cMLCK expression in the patient with *MYLK3* mutation is not secondary to heart failure, but due to a pathological consequence of mutated *MYLK3*, we need more heart samples to reach conclusion. To elucidate the mechanical linkage between gene mutation and heart phenotype, further studies using disease models such as zebrafish, mice, and induced pluripotent stem cells from patients are needed. Also, studies of larger multiethnic cohorts are necessary to validate the role of *MYLK3* mutations in DCM pathogenesis and to examine specific genotype−phenotype associations.

In conclusion, we have first identified *MYLK3* mutations associated with DCM in humans. This *MYLK3*-associated subtype appears relatively responsive to medical treatment, and our findings have important implications for the prognosis and treatment of DCM patients and their family members. Non-symptomatic *MYLK3* mutation-carriers in a pedigree with familial DCM could profit from early genetic diagnosis, and preemptive pharmacological treatment from an early disease stage, thereby contributing to a better prognosis for such DCM patients. We also suggest that chemical modulation of cMLCK activity could be a potential therapeutic strategy for cardiomyopathy.

## Materials and Methods

### Subjects

The study protocol followed the ethical guidelines of the Declaration of Helsinki and was approved by the ethics committees of the Tokyo Women’s Medical University and the University of Tokyo. All subjects provided written informed consent and all experiments were performed in accordance with relevant guidelines and regulations. DCM diagnosis was based on the presence of LV dilatation and dysfunction in the absence of heart disorders such as hypertensive heart disease, primary valvular disease, and coronary artery disease. Genomic DNA was extracted from whole blood by standard procedures.

### WES, targeted sequencing, and data analysis

Exome capture was performed with an Agilent SureSelectXT Human All Exon Kit V6 and a sequence library was prepared according to the manufacturer’s protocol for Illumina paired-end sequencing (Illumina, San Diego, CA, USA). Exome sequencing was performed on an Illumina HiSeq. 2500 using 100-bp paired-end reads. For target sequencing, we designed a panel of 95 genes (Supplementary Table 2) associated with DCM or other inherited cardiovascular diseases using SureDesign for Haloplex technology (Agilent Technologies, Santa Clara, CA, USA) that covered 99.4% of the exons of the target genes. Targeted sequencing was performed on an Illumina HiSeq. 2500 system in rapid-run mode, producing 150-bp paired-end reads.

For data analysis, filtered reads were mapped to the GRCh37/hg19 human reference genome with BWA-MEM^26^. The initial detection of variants was carried out using Surecall 3.0 (Agilent Technologies), which incorporates SAMtools^27^ and SNPPET (Agilent Technologies). We excluded intronic variants and synonymous mutations, as well as variants with minor allele frequency >0.01% in any freely accessible, ethnically-matched population database, such as the 1000 Genomes Project^28^, Exome Aggregation Consortium^29^, Human Genetic Variation Database (http://www.hgvd.genome.med.kyoto-u.ac.jp/index.html)^30^, Tommo database^31^, and our internal database of more than 800 exomes. Variants were also annotated with information from the Genome Aggregation database (gnomAD; http://gnomad.broadinstitute.org). Finally, mapped reads and called mutations were confirmed by inspection using the Integrative Genomics Viewer^32^. Deleterious variants were predicted *in silico* using a CADD score^33^, with a cutoff of 10. Function and tissue expression for each gene was investigated using database of pubmed (https://www.ncbi.nlm.nih.gov/pubmed), Mouse Genome Informatics (MGI; http://www.informatics.jax.org)^34^, GeneCards (http://www.genecards.org)^35^, and our internal database. The positions of *MYLK3* mutations are based on NCBI RefSeq NM_182493.

### MYLK3 gene sequencing

All exons and flanking introns of *MYLK3* were amplified by PCR using the primer pairs listed in Supplementary Table 5. The products were then sequenced with a BigDye Terminator V3.1 kit (Applied Biosystems, Foster City, CA, USA).

### DNA constructs

A full-length cDNA clone of human *MYLK3* (NM_182493) with an N-terminal His Tag and an additional 57 C-terminal bases was purchased from Eurofins (Tokyo, Japan). Both p.*820Sext*19 and p.L627fs*41 mutations of *MYLK3* were induced by site-directed mutagenesis using a QuikChange XL Site-Directed Mutagenesis Kit (Agilent Technologies). Wild-type and mutant constructs were subcloned into the pShuttle-CMV vector^36^ (a gift from B. Vogelstein). The specific primers for mutagenesis are shown in Supplementary Table 6. Introduction of the correct mutations was verified by DNA sequencing. A full-length cDNA clone of human *MYL2* (NM_000432) with an C-terminal Myc-FLAG-Tag was obtained from Origene (Rockville, MD, USA).

### Cell culture studies of HEK293T cells

HEK293T cells were grown in Dulbecco’s modified Eagle’s medium supplemented with 10% fetal bovine serum and L-glutamine at 37 °C under a 5% CO_2_ atmosphere. The cells were transfected using Lipofectamine 3000 (Invitrogen, Carlsbad, CA, USA) following the manufacturer’s instructions. In brief, the cells were seeded to reach 60−70% confluence at 24 h later, and then transfected with wild-type or mutated *MYLK3* cDNA constructs. Total cell lysates were extracted at 36 h after transfection and analyzed by immunoblotting. For the protein stability experiments, medium containing 100 µg ml^−1^ of cycloheximide (Sigma) was added at 16 h after transfection and total cell lysates were extracted at indicated time points.

### Protein extraction and immunoblot analysis

The cells were lysed in buffer containing 10 mM Tris-HCl, 150 mM NaCl, 5 mM EDTA, 1% Triton X-100, 1% sodium deoxycholate, 0.1% sodium dodecyl sulfate, complete protease inhibitor cocktail, and PhoStop EASYpack (Roche). After centrifugation at 15,000 rpm for 20 min at 4 °C, the supernatants were recovered and total protein concentrations were measured using a bicinchoninic acid assay (Pierce BCA Protein Assay Kit; Thermo Scientific). For immunoblot analysis, extracted proteins were separated on 4–15% Mini-PROTEAN TGX precast gradient gels (Bio-Rad) and transferred onto nitrocellulose membranes. The membranes were blocked with 2% nonfat dry milk powder in Tris-buffered saline plus 0.05% Tween and incubated overnight at 4 °C with an anti-MYLK3 antibody (1:500; Novus Biologicals, Littleton, CO, USA; cat# NBP1–86648) recognizing residues 27−144 of human cMLCK and an anti-β-actin antibody (1:5000; Sigma cat# A5441) as a loading control. Primary antibodies were detected with horseradish peroxidase (HRP)-conjugated species-specific secondary antibodies (GE Healthcare) and ECL plus (Thermo Scientific) using a LAS 3000 analyzer (GE Healthcare). Human cardiac tissue and non-transfected HEK293T cells were used as controls. Immunoblot band intensities were measured using ImageJ software (National Institutes of Health).

### Analysis of the MLC2 phosphorylation

For the phosphorylation analysis, HEK293T cells were co-transfected with wild-type or mutated *MYLK3* and *MYL2* cDNA constructs. The cells were lysed at 16 h after transfection in buffer containing 50 mM Tris-HCl, 120 mM NaCl, 0.5% NP-40, complete EDTA free protease inhibitor cocktail, and PhoStop EASYpack and proteins were extracted as described above. The extracted proteins were separated by SDS-PAGE on 12.5% polyacrylamide gels containing 50 μmol/L Phos-tag (Wako Pure Chemical Industries, Japan) and 100 μmol/L ZnCl_2._ After electrophoresis, the gel was washed three times in transfer buffer containing 10 mM EDTA for 10 min. The proteins were then transferred onto nitrocellulose membranes. The membranes were blocked with 5% nonfat dry milk powder in Tris-buffered saline plus 0.05% Tween and incubated 1 h at room temperature with an anti-FLAG antibody (1:100; Sigma cat# A8592). Detection and measurement of band intensity were performed as described above. MLC2 phosphorylation ratio was calculated as follows: MLC2 phosphorylation = (phosphorylated MLC2)/(non-phosphorylated MLC2+ phosphorylated MLC2)

### Immunohistochemistry

Immunohistochemistry was performed on paraffin-embedded 4 µm-thick tissue sections obtained from an endomyocardial biopsy of the proband in family A. Deparaffinization and rehydration were performed using a standard procedure with xylene and ethanol. To block non-specific binding, the sections were incubated for 5 min with normal horse serum (Vector Laboratories, La Jolla, CA, USA; cat# PK-7200). The samples were incubated for 1 h at room temperature with an anti-MYLK3 antibody (1:500; Novus Biologicals; cat# NBP1-86648) and an anti-muscle actin antibody (1:200; Dako; cat# M0635). The slides were incubated with biotinylated anti-mouse and anti-rabbit IgG secondary antibodies for 30 min and with the VECTASTAIN ABC reagent (Vector Laboratories; cat# PK-7200) for 30 min. Then, the slides were visualized with the ImmPACT Diaminobenzidine (DAB) Peroxidase (HRP) Substrate (Vector Laboratories; cat# SK-4105) followed by counterstaining with hematoxylin. For comparison, heart tissues of right ventricle from a 29-year-old male with no evidence of cardiac disorders and endomyocardial biopsy specimens from a 21-year-old male DCM patient with an *RBM20* mutation (p.R634W), and a 50-year-old female DCM patient with a *BAG3* truncation mutation (p.H166fs*6) in our cohort were used. They were confirmed not to have any *MYLK3* mutations by Sanger sequencing. The positions of the *RBM20* and *BAG3* mutations are based on NCBI RefSeq NM_001134363 and NM_004281, respectively.

### Statistical analysis

Data are shown as the mean ± standard error of the mean (SEM). Statistical analysis was performed with SAS software JMP version 11.0. Group means were compared by Student’s t-test, and *P* < 0.05 was considered statistically significant.

### Data availability

Genetic data have been deposited at the Japanese Genotype−phenotype Archive (http://trace.ddbj.nig.ac.jp/jga), which is hosted by the DNA Data Bank of Japan, under accession number JGAS00000000110.

## Electronic supplementary material

Supplementary materials
