# Supplementary material for: Identification of MYLK3 mutations in familial dilated cardiomyopathy
Source: Sci Rep. 2017 Dec 13;7:17495. doi: 10.1038/s41598-017-17769-1 (PMC5727479; doi:10.1038/s41598-017-17769-1)
Supplement: Supplementary file 1 — Supplementary materials [file 41598_2017_17769_MOESM1_ESM.pdf]

Supplementary Materials

Identification of *MYLK3* mutations in familial dilated cardiomyopathy

Takashige Tobita[1], Seitaro Nomura[2,3], Hiroyuki Morita[2], Toshiyuki Ko[2], Takanori Fujita[3], Haruhiro Toko[2], Kenta Uto[1], Nobuhisa Hagiwara[1], Hiroyuki Aburatani[3], and Issei Komuro[2]

[1]Department of Cardiology, Tokyo Women's Medical University; [2]Department of Cardiovascular Medicine, Graduate School of Medicine, The University of Tokyo; [3]Genome Science Division, Research Center for Advanced Science and Technology, The University of Tokyo

Correspondence: Hiroyuki Aburatani and Issei Komuro

Address for correspondence:

Full name: Hiroyuki Aburatani

Postal address: 4-6-1 Komaba, Meguro-ku, Tokyo 153-8904, JAPAN

Telephone: +81-3-5452-5352 Fax number: +81-3-5452-5355

E-mail: haburata-tky@umin.ac.jp

Full name: Issei Komuro

Postal address: 7-3-1 Hongo, Bunkyo-ku, Tokyo 113-8655, JAPAN

Telephone: +81-3-5800-6526 Fax number: +81-3-3815-2087

E-mail: komuro-tky@umin.ac.jp

Suplementary Table 1. Variants shared by three DCM patietns in family A. Expression levels in cardiomyocytes for each gene were annotated using our internal database. Data of tissues showing specific expression was obtained from GeneCards. N/A, not applicable.

Gene	Chrom	Pos	Mutation type	Nucleotide Change	Amino Acid Change	RefSeq	ExAC east (count)	gnomAD (count)	CADD	Reported to be associated with dilated cardiomyopathy in human	Mouse mutant phenotypes (MGI)	Expression levels (RPKM) in cardiomyocytes	Tissues showing specific expression
ATRN	20	3451843	missense	c.89A>C	p.H30P	NM_139321	absent	absent	3.19	-	adipose tissue, behavior/neurological, growth/size/body, integument, mortality/aging, muscle, nervous system, pigmentation	4.0	N/A
CCDC135	16	57732922	missense	c.364G>A	p.E122K	NM_032269	absent	3	34.00	-	-	0.0	N/A
CRTAM	11	122724744	missense	c.440A>G	p.K147R	NM_019604	absent	1	22.60	-	hematopoietic system, immune system	0.0	brain
EMID1	22	29602092	missense	c.65C>T	p.A22V	NM_133455	absent	absent	26.00	-	homeostasis/metabolism	0.0	spleen
GOLIM4	3	167747667	missense	c.1334G>A	p.R445Q	NM_014498	absent	1	15.38	-	-	1.0	N/A
ITLN2	1	160924228	missense	c.28A>G	p.R10G	NM_080878	absent	absent	8.80	-	-	N/A	small Intestine
KIAA1009	6	84922701	missense	c.545A>G	p.N182S	NM_014895	absent	absent	0.60	-	-	N/A	N/A
LIPJ	10	90362400	missense	c.791G>A	p.S264N	NM_001010939	absent	absent	7.41	-	-	N/A	testis
METTL24	6	110636655	missense	c.447C>G	p.S149R	NM_001123364	absent	56	24.80	-	behavior/neurological, growth/size/body, hearing/vestibular/ear	N/A	N/A
MYLK3	16	46741617	read through	c.2459A>C	p.*820Sext*19	NM_182493	absent	absent	18.79	-	cardiovascular system, homeostasis/metabolism, mortality/aging, muscle	66.0	heart
PCDHA7	5	140216274	missense	c.2306T>C	p.L769P	NM_018910	absent	absent	26.00	-	-	0.0	brain
QTRT1	19	10812222	missense	c.86G>A	p.R29Q	NM_031209	absent	5	34.00	-	cellular, homeostasis/metabolism	3.7	N/A
SLC5A2	16	31496064	missense	c.197C>T	p.P66L	NM_003041	absent	2	27.50	-	adipose tissue, behavior/neurological, growth/size/body, homeostasis/metabolism, immune system, renal/urinary system, skeleton	0.0	kidney, testis
SORCS1	10	108431109	missense	c.2075T>C	p.M692T	NM_001013031	absent	absent	28.10	-	homeostasis/metabolism, nervous system	0.0	nerve, brain
SSPO	7	149474375	frameshift	c.419_420insC	p.C140fs	NM_198455	absent		9.72	-	-	0.0	brain
SYNE3	14	95884350	missense	c.2741G>A	p.R914Q	NM_152592	absent	11	16.83	-	-	N/A	adipose
TERF2	16	69418502	missense	c.587G>A	p.R196K	NM_005652	absent	absent	23.90	-	cellular, liver/biliary system, mortality/aging	2.7	N/A
TMEM128	4	4247954	missense	c.142C>T	p.L48F	NM_032927	absent	absent	10.45	-	-	7.9	N/A
UBN2	7	138978680	missense	c.3947C>T	p.T1316M	NM_173569	absent	495	25.20	-	-	1.7	N/A

Supplementary Table 2. Genes analyzed by targeted sequencing.

	Gene symbol	Official Full Name
**Cardiomyopathy reated genes**	ABCC9	ATP-binding cassette sub-family C member 9
	ACTC1	Actin alpha cardiac muscle 1
	ACTN2	Actinin alpha 2
	ANKRD1	Ankyrin repeat domain 1
	BAG3	BCL2-associated athanogene 3
	CALR3	Calreticulin 3
	CAV3	Caveolin 3
	CRYAB	Crystallin alpha B
	CSRP3	Cysteine and glycine-rich protein 3
	DES	Desmin
	DMD	Dystrophin
	DSC2	Desmocollin 2
	DSG2	Desmoglein 2
	DSP	Desmoplakin
	EMD	Emerin
	EYA4	EYA transcriptional coactivator and phosphatase 4
	FXN	Frataxin
	GLA	Galactosidase alpha
	ILK	Integrin linked kinase
	JPH2	Junctophilin 2
	JUP	Junction plakoglobin
	LAMP2	Lysosomal-associated membrane protein 2
	LDB3	LIM domain binding 3
	LMNA	Lamin A/C
	MYBPC3	Myosin binding protein C, cardiac
	MYH6	Myosin, heavy chain 6, cardiac muscle, alpha
	MYH7	Myosin, heavy chain 7, cardiac muscle, beta
	MYL2	Myosin light chain 2
	MYL3	Myosin light chain 3
	MYOM1	Myomesin 1
	MYOZ2	Myozenin 2
	MYPN	Myopalladin
	NEBL	Nebulette
	NEXN	Nexilin F-actin binding protein
	PDLIM3	PDZ and LIM domain 3
	PKP2	Plakophilin 2
	PLN	Pphospholamban
	PRKAG2	Protein kinase AMP-activated non-catalytic subunit gamma 2
	PTPN11	Protein tyrosine phosphatase, non-receptor type 11
	RAF1	Raf-1 proto-oncogene, serine/threonine kinase
	RBM20	RNA binding motif protein 20
	RYR2	Ryanodine receptor 2
	SCN5A	Sodium voltage-gated channel alpha subunit 5
	SGCD	Sarcoglycan delta
	TAZ	Tafazzin
	TCAP	Titin-cap
	TGFB3	Transforming growth factor beta 3
	TMEM43	Transmembrane protein 43
	TMPO	Thymopoietin
	TNNC1	Troponin C1, slow skeletal and cardiac type
	TNNI3	Troponin I3, cardiac type
	TNNT2	Troponin T2, cardiac type
	TPM1	Tropomyosin 1 (alpha)
	TTN	Titin
	TTR	Transthyretin
	TXNRD2	Thioredoxin reductase 2

	VCL	Vinculin
**Arrhythmia related genes**	AKAP9	A-kinase anchoring protein 9
	ANK2	Ankyrin 2, neuronal
	CACNA1C	Calcium voltage-gated channel subunit alpha1 C
	CACNB2	Calcium voltage-gated channel auxiliary subunit beta 2
	CASQ2	Calsequestrin 2
	GPD1L	Glycerol-3-phosphate dehydrogenase 1-like
	KCNE1	Potassium voltage-gated channel subfamily E regulatory subunit 1
	KCNE2	Potassium voltage-gated channel subfamily E regulatory subunit 2
	KCNE3	Potassium voltage-gated channel subfamily E regulatory subunit 3
	KCNH2	Potassium voltage-gated channel subfamily H member 2
	KCNJ2	Potassium voltage-gated channel subfamily J member 2
	KCNQ1	Potassium voltage-gated channel subfamily Q member 1
	SCN1B	Sodium voltage-gated channel beta subunit 1
	SCN3B	Sodium voltage-gated channel beta subunit 3
	SCN4B	Sodium voltage-gated channel beta subunit 4
	SNTA1	Syntrophin alpha 1
**Noonan syndrome related genes**	BRAF	B-Raf proto-oncogene, serine/threonine kinase
	CBL	Cbl proto-oncogene
	HRAS	HRas proto-oncogene, GTPase
	KRAS	KRAS proto-oncogene, GTPase
	MAP2K1	Mitogen-activated protein kinase kinase 1
	MAP2K2	Mitogen-activated protein kinase kinase 2
	NF1	Neurofibromin 1
	NRAS	neuroblastoma RAS viral oncogene homolog
	RIT1	RAS like without CAAX 1
	SHOC2	SHOC2, leucine rich repeat scaffold protein
	SOS1	SOS Ras/Rac guanine nucleotide exchange factor 1
	SPREED1	Sprouty related EVH1 domain containing 1
**Marfan syndrome related genes**	ACTA2	Actin, alpha 2, smooth muscle, aorta
	CBS	Cystathionine-beta-synthase
	COL3A1	Collagen type III alpha 1 chain
	FBN1	Fibrillin 1
	FBN2	Fibrillin 2
	MYH11	Myosin, heavy chain 11, smooth muscle
	SLC2A10	Solute carrier family 2 member 10
	SMAD3	SMAD family member 3
	TGFBR1	Transforming growth factor beta receptor 1
	TGFBR2	Transforming growth factor beta receptor 2

Supplementary Table 3. Variants shared by two DCM patietns in family B. Expression levels in cardiomyocytes for each gene were annotated using our internal database. Data of tissues showing specific expression was obtained from GeneCards. N/A, not applicable.

Gene	Chrom	Pos	Mutation type	Nucleotide Change	Amino Acid Change	RefSeq	ExAC east (count)	gnomAD (count)	CADD	Reported to be associated with dilated cardiomyopathy in human	Mouse mutant phenotypes (MGI)	Expression levels (RPKM) in cardiomyocytes	Tissues showing specific expression
A2M	12	9258864	missense	c.1072C>T	p.H358Y	NM_000014	absent	absent	0.001	-	-	0.0	lung, liver
APLNR	11	57003769	missense	c.710T>C	p.I237T	NM_005161	absent	absent	2.725	-	behavior/neurological, cardiovascular system, embryo, growth/size/body, homeostasis/metabolism, mortality/aging, muscle, renal/urinary system, reproductive system, respiratory system	0.0	brain
ARHGAP35	19	47425068	missense	c.3136C>A	p.P1046T	NM_004491	absent	absent	9.196	-	cellular, embryo, growth/size/body, mortality/aging, nervous system, pigmentation, renal/urinary system, vision/eye	2.9	N/A
CCER2	19	39401445	missense	c.469C>T	p.R157W	NM_001243212	absent	16	10.85	-	-	N/A	N/A
CIITA	16	11016046	missense	c.3172G>A	p.D1058N	NM_000246	absent	3	27.1	-	endocrine/exocrine glands, hematopoietic system, homeostasis/metabolism, skeleton	0.0	spleen
CLMN	14	95660899	nonsense	c.2743C>T	p.R915*	NM_024734	absent	2	41	-	-	0.0	N/A
CTTNBP2	7	117422917	missense	c.2371G>A	p.E791K	NM_033427	absent	3	19.39	-	skeleton	0.0	N/A
GEMIN5	5	154271108	missense	c.3955G>C	p.D1319H	NM_015465	absent	1	23.4	-	-	1.0	N/A
KRT6B	12	52845486	missense	c.377C>A	p.G126D	NM_005555	absent	165	20.5	-	behavior/neurological, craniofacial, digestive/alimentary system, growth/size/body, integument, mortality/aging	0.0	esophagus, skin, minor salivary gland, cervix, vagina
LPHN2	1	82436119	missense	c.2804C>T	p.S935L	NM_012302	absent	absent	25.1	-	behavior/neurological, mortality/aging	3.4	N/A
MAP2K2	19	4101118	missense	c.604G>A	p.V202M	NM_030662	absent	2	29.3	-	adipose tissue, behavior/neurological, growth/size/body	14.5	N/A
MCC	5	112363165	missense	c.2894C>T	p.A965V	NM_001085377	absent	absent	26.4	-	-	0.1	ovary
MIPEP	13	24460473	missense	c.362T>G	p.L121W	NM_005932	absent	absent	27	-	-	23.5	N/A
MUS81	11	65633499	nonsense	c.1632C>G	p.Y544*	NM_025128	absent		37	-	cardiovascular system, cellular, homeostasis/metabolism, immune system, mortality/aging, respiratory system, neoplasm	4.1	N/A
MYLK3	16	46761176	frameshift	c.1879_1885 del	p.L627fs*41	NM_182493	absent	absent	35	This study	cardiovascular system, homeostasis/metabolism, mortality/aging, muscle	66.0	heart
PEX6	6	42934364	missense	c.1993G>C	p.G665R	NM_000287	absent	absent	20.4	-	-	8.2	
PRAMEF4	1	12942207	missense	c.343T>C	p.W115R	NM_001009611	absent	absent	22.7	-	-	N/A	N/A
PTPRR	12	71077941	missense	c.1463T>A	p.I488N	NM_002849	absent	absent	32	-	behavior/neurological	0.1	brain
RAPGEF2	4	160277148	missense	c.4312G>T	p.V1438F	NM_014247	absent	absent	18.27	-	cardiovascular system, cellular, digestive/alimentary system, embryo, growth/size/body, hematopoietic system, integument, liver/biliary system, mortality/aging, nervous system	1.4	N/A
SETMAR	3	4355207	missense	c.782A>G	p.D261G	NM_006515	absent	absent	26	-	behavior/neurological, growth/size/body, homeostasis/metabolism, nervous system	0.3	N/A
SLC35A4	5	139947565	missense	c.811A>G	p.M271V	NM_080670	absent	1	19.94	-	-	10.0	N/A
TM2D1	1	62160394	missense	c.488T>C	p.I163T	NM_032027	absent	absent	27.5	-	-	21.4	N/A
TMEM63C	14	77706894	missense	c.1007C>T	p.P336L	NM_020431	absent	absent	8.576	-	-	0.0	pituitary, brain
TXNDC16	14	52899296	missense	c.2204C>G	p.P735R	NM_020784	absent	absent	23.6	-	-	1.8	N/A
USP44	12	95927917	nonsense	c.116G>A	p.W39*	NM_032147	absent	absent	37	-	cellular, liver/biliary system, respiratory system, neoplasm	0.0	testis, brain
VEZT	12	95660150	missense	c.452T>A	p.F151Y	NM_017599	absent	absent	29.3	-	behavior/neurological, cellular, embryo, homeostasis/metabolism, mortality/aging, reproductive system	6.0	N/A
ZNF212	7	148950769	missense	c.751C>A	p.L251I	NM_012256	absent	absent	14.07	-	-	N/A	N/A
ZNF676	19	22364145	missense	c.374G>T	p.C125F	NM_001001411	absent	absent	0.007	-	-	N/A	thyroid, testis
ZXDC	3	126180569	missense	c.1936C>T	p.P646S	NM_001040653	absent	absent	0.011	-	-	2.1	N/A

Supplementary Table 4. Variants shared by only two siblings in family A. Expression levels in cardiomyocytes for each gene were annotated using our internal database. Data of tissues showing specific expression was obtained from GeneCards. N/A, not applicable.

[1] Ortiz-Genga, M. F. *et al.* Truncating FLNC Mutations Are Associated With High-Risk Dilated and Arrhythmogenic Cardiomyopathies. *J Am Coll Cardiol*. **68**, 2440-2451 (2016).

[2] Reinstein, E. *et al*. Congenital dilated cardiomyopathy caused by biallelic mutations in Filamin C. *Eur J Hum Genet*. **24**, 1792-1796 (2016).

Gene	Chrom	Pos	Mutation type	Nucleotide Change	Amino Acid Change	RefSeq	ExAC east (count)	gnomAD (count)	CADD	Reported to be associated with dilated cardiomyopathy in human	Mouse mutant phenotypes (MGI)	Expression levels (RPKM) in cardiomyocytes	Tissues showing specific expression
ANO7	2	242151518	missense	c.1733G>A	p.R578H	NM_001001891	absent	10	31.00	-	-	0.0	prostate, colon, stomach, small Intestine
ATP11B	3	182607208	missense	c.2854A>G	p.K952E	NM_014616	absent	absent	25.40	-	-	1.8	whole blood
CCDC61	19	46509892	missense	c.307C>T	p.R103C	NM_001267723	absent	3	31.00	-	-	0.6	N/A
CELF4	18	34844647	missense	c.1239G>C	p.Q413H	NM_020180	absent	2	23.50	-	behavior/neurological, embryo, endocrine/exocrine glands, growth/size/body, homeostasis/metabolism, immune system, liver/biliary system, mortality/aging, muscle, nervous system, vision/eye	0.0	brain
FLNC	7	128480109	nonsense	c.1444C>T	p.R482*	NM_001458	absent	absent	38.00	[1, 2]	behavior/neurological, growth/size/body, integument, mortality/aging, muscle, respiratory system	12.7	muscle, heart
ITSN2	2	24494664	missense	c.2228A>T	p.K743M	NM_147152	absent	5	15.20	-	behavior/neurological, hematopoietic system, integument	1.5	N/A
LMAN2	5	176778642	missense	c.7G>A	p.A3T	NM_006816	absent	absent	23.30	-	-	8.2	N/A
LMBRD1	6	70506710	missense	c.64C>G	p.L22V	NM_018368	absent	absent	21.20	-	cardiovascular system, cellular, mortality/aging, muscle	5.3	N/A
MAPK12	22	50691871	missense	c.1063C>T	p.R355W	NM_002969	absent	1	23.70	-	cardiovascular system, cellular	3.4	muscle
MMAA	4	146572283	missense	c.803G>T	p.G268V	NM_172250	absent	2	32.00	-	-	7.5	liver
MYO1G	7	45009012	missense	c.1574C>T	p.T525M	NM_033054	absent	6	22.50	-	hematopoietic system, immune system	0.0	whole blood
PODNL1	19	14043690	missense	c.1367G>A	p.R456Q	NM_024825	absent	12	6.66	-	-	0.0	nerve, artery
SYT5	19	55684980	missense	c.1033G>A	p.V345M	NM_003180	absent	absent	25.00	-	mortality/aging, nervous system	0.0	brain, pituitary
TMEM119	12	108985951	missense	c.209A>C	p.Q70P	NM_181724	absent	absent	0.98	-		0.0	N/A

Supplementary Table 5. Primers for PCR and Sanger sequencing

MYLK3	Primer (Forward)	Primer (Reverse)
exon 1	ATGCCTCCAGCTCTCCAAAC	TCTGTCAGCTACGGAGGACA
exon 2	CCGAGGTGTTCAAGGGTCAA	CTGGGCGCCTCCTCTTTTAA
exon 3	ACTCTAAGCTCCCAGAGGCA	GCCTTGTGCAGATTCCTTGC
exon 4	ATGTTCCCGACTCTTGGACG	GTCAATGATGGCAGCAGTGG
exon 5	GGCTCTCGTGTCCTCTCAAG	CCTGGGTTTGAGTGCTGTCT
exon 6	CTACAGAGCTGTGCCTGGAC	GGATGTTCCCTGGGATGTGG
exon 7	CCTGCAGGTCAAATCAGGCT	GTCACACTCAGGCCCTTTGT
exon 8	CCAAGAGGCAGCAAGAGTGG	CTTGAACCCAGGAGACGGAG
exon 9	AAGTCAAAGGCCACCAGCTT	CCGGCTTCTCTTGAGGACTG
exon 10	ATACCCTTCCCTGGCTCCTT	ATGCCTGGAGGAGGAAGACT
exon 11	CTTCACGGTCAGCAGTCCTG	GCATACCAAGTTGGGCCCTA
exon 12	ACCATGATCATGCCACTATACTCC	ACATTTTGCTGACGACCTTCT
exon 13	CGACAGATGCAAAGTACCTACCT	GGGCAGGATGATGTATCTTCCC

Supplementary Table 6. Primers for mutagenesis

Mutation	Primer (Forward)	Primer (Reverse)
p.*820Sext*19	GAAATTTCCAACTTCTCCCTCAT CTTCAACTCTGC	GAGCAGCAGAGTTGAAGATGAGGGA GAAGTTGGAA
p.L627fs*41	TCTGTGAGGGTGTGCATTACAGC ACTACATCCTGC	CAGGTGCAGGATGTAGTGCTGTAAT GCACACCCTC

Supplementary Fig. 1

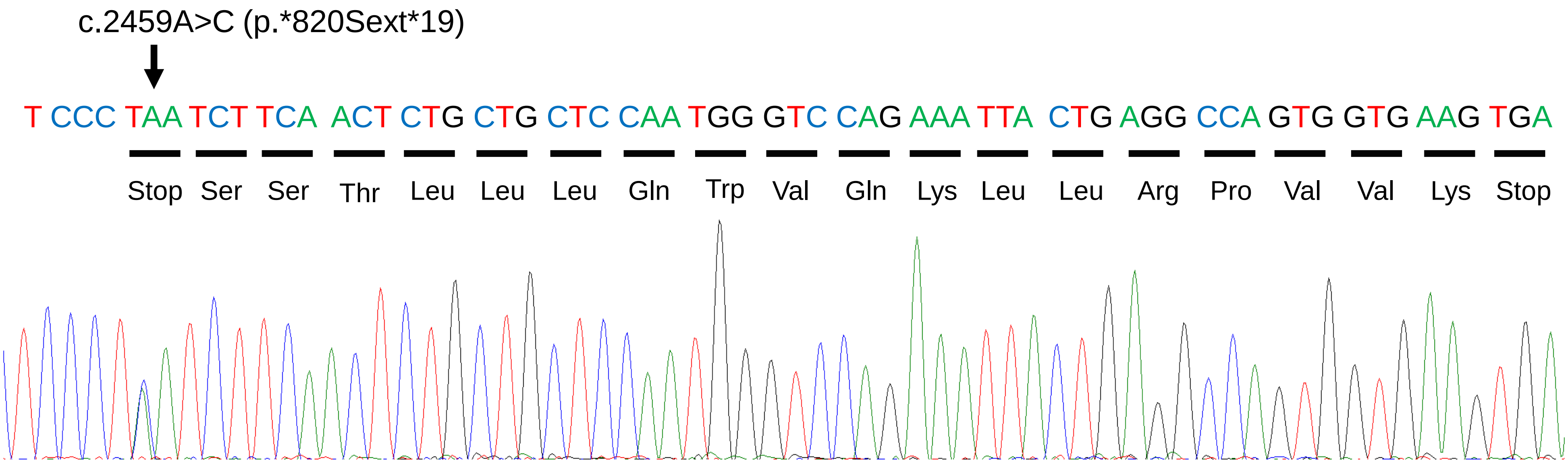

Supplementary Fig. 2

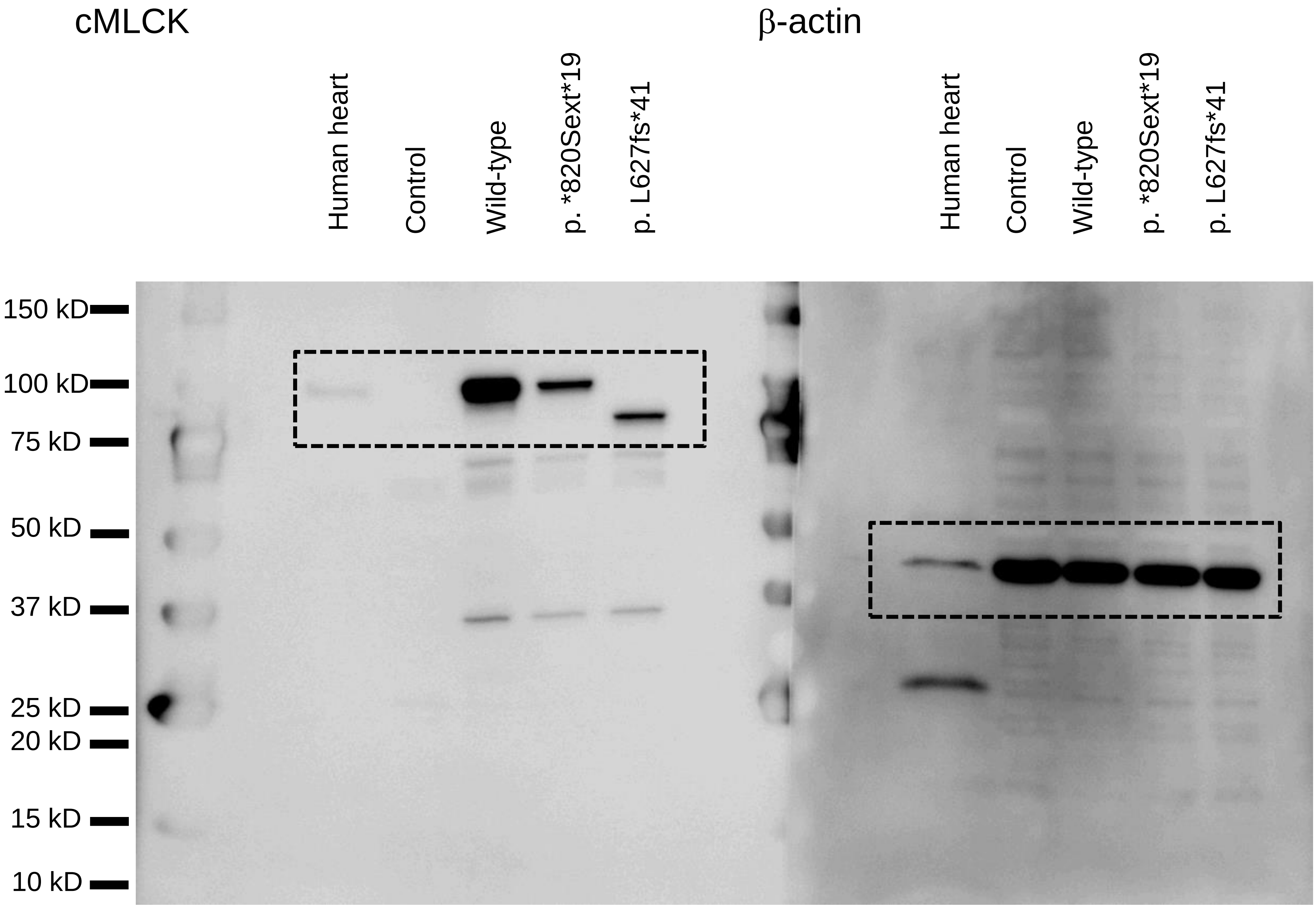

Supplementary Fig. 3

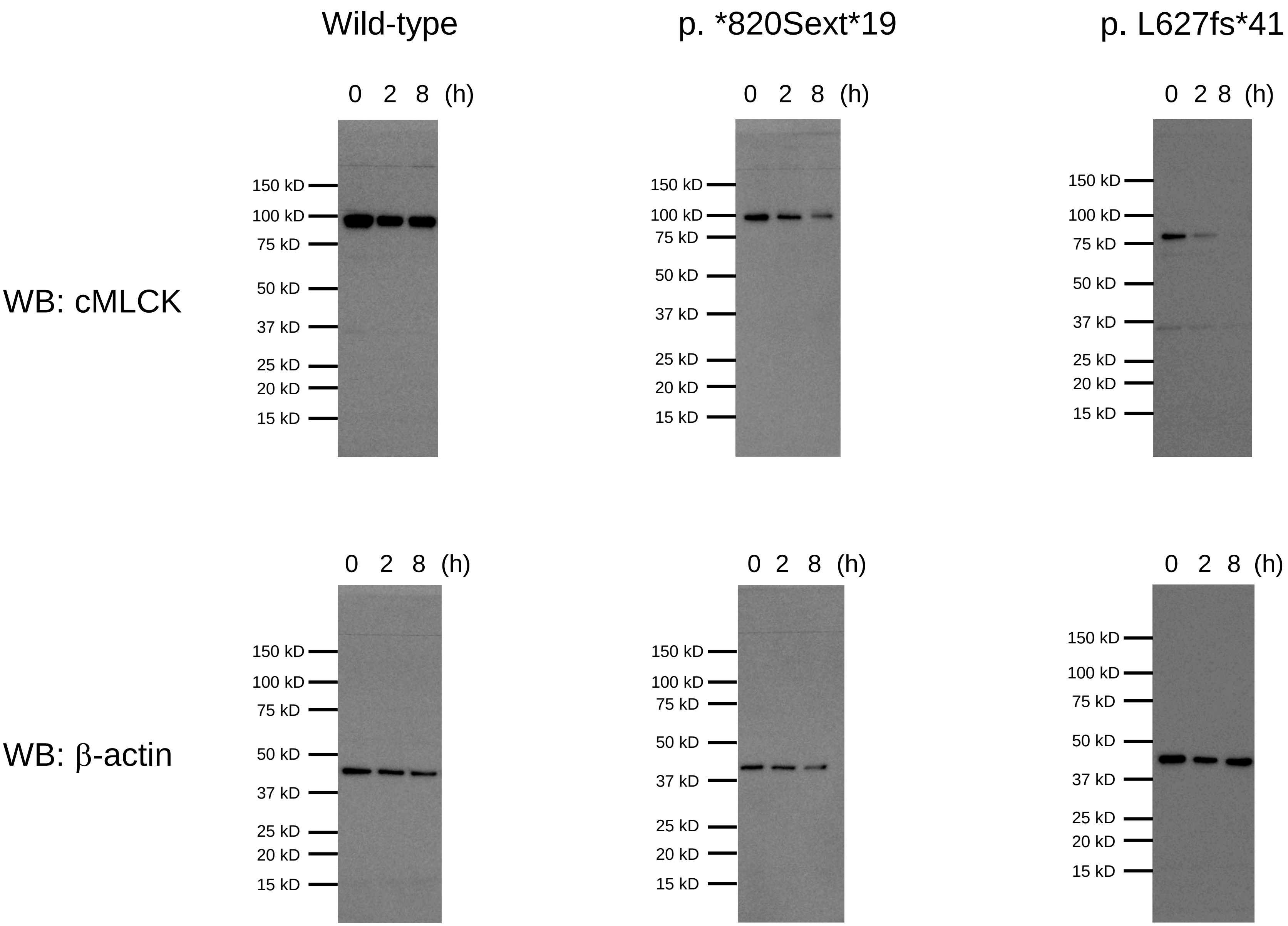

Supplementary Fig. 4

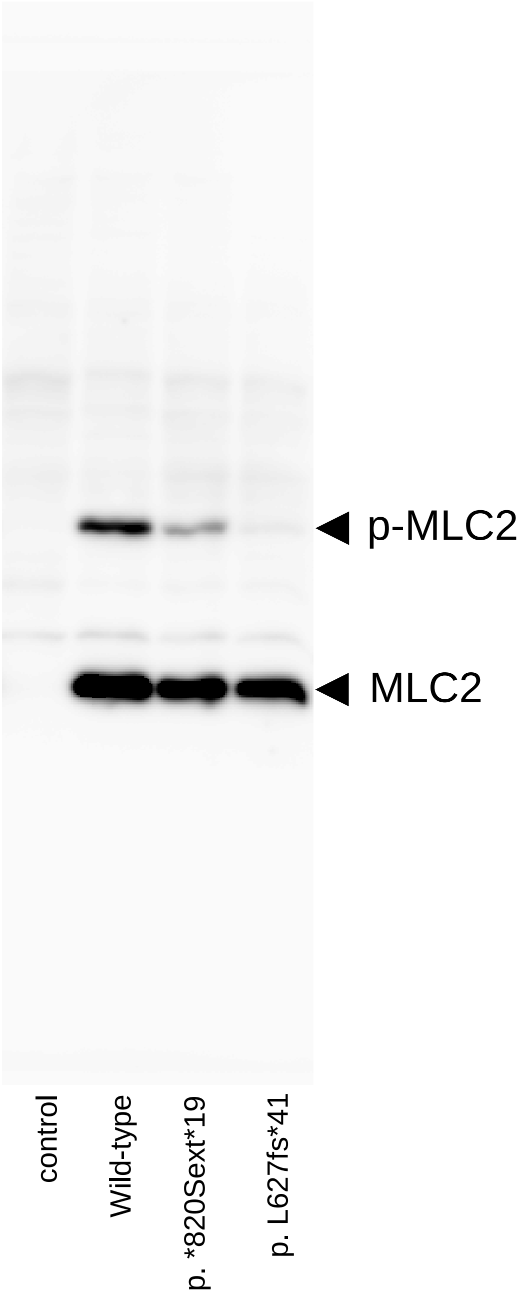

Supplementary figure legends

Supplementary Figure 1. Sanger sequencing analysis verified the presence of a *MYLK3* read-through mutation resulting in a protein product with a 19-amino acid C-terminal extension.

Supplementary Figure 2. Uncropped western blots referring to Figure 4a.

Supplementary Figure 3. Uncropped western blots referring to Figure 4c.

Supplementary Figure 4. Uncropped western blots referring to Figure 4d.
